# P12 Assessing the clinical utility of antibiotics for urinary tract infections in India: findings from a questionnaire survey

**DOI:** 10.1093/jacamr/dlad077.016

**Published:** 2023-08-02

**Authors:** Narmada Prasad Gupta, Percy Chibber, Umesh Oza, Anant Kumar, D Ramesh, Sanjay Pandey, N Mallikarjuna Reddy, Bibash Kundu, Rajesh Taneja, Ganesh Kamath

**Affiliations:** Medanta, The Medicity, Gurgaon, India; Sir H. N. Reliance Foundation Hospital and Research Centre, Mumbai, India; P. D. Hinduja Hospital, Mumbai, India; Max Hospital, Delhi, India; M. S. Ramaiah Hospital, Bangalore, India; Kokilaben Hospital, Mumbai, India; Citizens Speciality Hospital, Hyderabad, India; Calcutta Medical Research Institute, Kolkata, India; Apollo Hospital, Delhi, India; Sundaram Medical Foundation, Chennai, India

## Abstract

**Background:**

We aimed to formulate a consensus for the therapeutic utilization of antibiotics based on contemporary evidence and the real-world experiences of the clinicians at the forefront of the care of patients with urinary tract infection (UTI).

**Methods:**

We developed customized technological response system for mapping exercise and panel of eminent specialists was convened. Prior consent was obtained, and the weblink of the questionnaire was provided. Sub-expert nationwide panel (*n*=397), across India (PRISM Survey group), rated their level of agreement with 11 questions with each item on a five-point Likert scale and 5 objective response questions. The consensus was pre-defined if the weighted mean score for the Likert scale was >100. Data were statistically analysed by GraphPad software version 9.5.0.

**Results:**

The mean years of experience were 9.2 years (SD ±10, 95% CI 8.2–10), and cumulative clinical experience was 3669 person-years. The predominant clinical practice setting of the respondents was a public hospital (58.6%). The highest agreement score in the decreasing rank order (of adjusted weighted mean score) for UTI were: antibiotic resistance is a major challenge (126.1), oral fosfomycin-based regimens are effective against MDR pathogens (123.4), is a good option for bacterial biofilm reduction (121.7), the potential to manage recurrent lower UTI (121.6), oral fosfomycin along with nitrofurantoin is been useful to manage MDR UTI (106.8), nitrofurantoin and fosfomycin are chosen as empirical treatment (103.7), fosfomycin has stood the test of time (120.2). Adjusted mean response scores (±SD, 95% CI) for consensus were: agree (73±9.4, 95% CI 66 to 79) followed by strongly agree (32±13, 95% CI 24 to 41), neither agree nor disagree (13±4.9, 95% CI 9.8 to 16), disagree (−6.5±4.7, 95% CI −9.7 to −3.4), strongly disagree (−0.86±0.9, 95% CI −1.5 to −0.26).

**Conclusions:**

In the era of MDR UTI, time-tested oral fosfomycin and nitrofurantoin can improve management of UTI without increasing the complexity.

